# The Evaluation and Modification of Standard Airway Assessment Tests for Virtual Anaesthetic Assessments: A Pilot Study

**DOI:** 10.3390/jcm14020342

**Published:** 2025-01-08

**Authors:** Wan Yen Lim, Sharon Gek Kim Ong, Jia Xin Chai, Rhommela Garis Duran, Ahmad Hamidi Mohammed Ali, John Ong

**Affiliations:** 1 Department of Anaesthesiology, Sengkang General Hospital, Singapore 544886, Singapore; 2 Department of Anaesthesiology, Singapore General Hospital, Singapore 169608, Singapore; 3 Duke-NUS Medical School, Singapore 169857, Singapore; 4 School of Clinical Medicine, University of Cambridge, Cambridge CB2 0SP, UK; jo401@cam.ac.uk

**Keywords:** airway assessment, telemedicine, anaesthesia, virtual consultation, upper lip bite test, Mallampati score, screening tools

## Abstract

**Background/Objectives:** Virtual preoperative anaesthetic assessments can significantly reduce healthcare costs and improve patient convenience. The challenge with virtual consults is often the airway assessments, which screen for potentially difficult airways (PDAs). The objective of this pilot study was to determine the reliability of standard airway screening tests for detecting PDAs when conducted virtually. **Methods:** An observational longitudinal study was conducted between July 2021 and April 2022 at a tertiary hospital in Singapore. We compared the Mallampati score (MS), upper lip bite test (ULBT), thyromental distance, mouth opening test, and neck movements in 94 patients, first during virtual assessments before surgery and subsequently at face-to-face preoperative assessments (gold standard) on the day of surgery by the same team of anaesthesiology trainees. Goodman and Kruskal’s gamma coefficient measured concordance between virtual and face-to-face assessment results. Logistic regression (LR) identified virtual predictors of PDAs in clinical practice. AUROC values informed tool performance. **Results:** LR showed that elevated virtual MS, virtual ULBT, and body mass index (BMI) were potential predictors of clinical PDAs. Termed the “MBBS”, this collective score showed good performance with a sensitivity of 95% and an AUROC of 0.79. Importantly, all screening tests performed poorly in virtual assessments when applied individually (sensitivity < 50%). **Conclusions:** In this pilot study, BMI combined with MS and ULBT could reliably detect PDAs during virtual airway assessments. The data herein support further large multi-centre studies to validate the MBBS for clinical use.

## 1. Introduction

Failed intubation and ventilation due to an unanticipated difficult airway is a leading cause of anaesthesia-associated morbidity and mortality worldwide [1]. Recent European Society of Anaesthesiologists and Intensive Care guidelines recommend a comprehensive pre-anaesthetic airway evaluation incorporating multiple tests for better predictive value and clinical utility [2]. The Mallampati score (MS), thyromental distance (TMD), upper lip bite test (ULBT), and mouth opening test (MOT) are the most commonly used airway assessment tests worldwide to screen for potentially difficult airways (PDAs) [3]. Internationally, for elective surgeries, these tests are traditionally performed face-to-face by trained anaesthesiologists during a preoperative visit to the anaesthesiology clinic. However, due to the rising demand for healthcare and the need for cost-efficient processes in healthcare systems, there has been a greater emphasis on transitioning some services to telemedicine after the COVID-19 pandemic [4,5]. Like many developed countries, Singapore has a large ageing population and rising healthcare costs. Therefore, we have explored novel initiatives to facilitate online anaesthesiology services. 

Virtual airway assessments have previously been suggested [6]. Reported high patient satisfaction rates with telemedicine were often attributed to an efficient and accurate pre-anaesthesia evaluation, which reduced both the time and the monetary costs associated with travel to a clinic, in the range of 24 to 137 min and USD 60 to USD 67 per patient, respectively, without increasing surgery cancellations [2,7,8,9]. In addition to healthcare costs and time savings (e.g., commute and patient waiting time), the transport-associated “carbon footprint” is also significantly reduced. For these reasons, a growing body of evidence reports the feasibility, effectiveness, and benefits of telemedicine in anaesthesiology [10]. 

Nonetheless, there are challenges limiting the integration of telemedicine into healthcare systems. These include limitations in technical infrastructure (e.g., adequate internet connectivity) and access to suitable equipment (e.g., webcams or smartphones with suitable specifications). As such, when conducting airway assessments virtually, the diagnostic accuracy of bedside airway screening tests remains to be determined [3,9,11]. Although the MS is widely incorporated into routine clinical airway assessment, additional factors in virtual assessments (e.g., poor lighting) can limit visual inspection of the oropharyngeal structures. Similarly, the ULBT has demonstrated high sensitivity and specificity in predicting difficult airways in the literature, but it is generally under-utilised with limited studies on virtual airway assessments [12]. Compared to the MS, the ULBT is easily performed during virtual consultation, but head-to-head virtual comparisons are also lacking. 

With recent technological advancements, digital cameras now provide better resolution and higher image capture speeds during video consultation, improving the quality of virtual airway assessments. Therefore, our primary objective was to determine the reliability of standard airway screening tests for detecting PDAs when used virtually. The secondary objective was to determine if modifications could be made to improve the predictive values of these tests in detecting PDAs from virtual assessments. These data could then justify further large-scale pragmatic clinical trials involving multiple centres.

## 2. Materials and Methods

We conducted an observational longitudinal study involving elective surgical patients who first underwent a preoperative virtual anaesthetic evaluation, followed by a routine face-to-face evaluation by the same group of anaesthesia trainee doctors on the day of their surgery, to assess variability between the two methods.

### 2.1. Ethics

Ethical approval was granted by the Singhealth Centralised Institutional Review Board (2021/2348). All patients provided informed and written consent.

### 2.2. Patient Selection

Inclusion criteria were (i) patients aged 21 to 65 years old, (ii) American Society of Anaesthesiology (ASA) physical status class 1 or 2, and (iii) patients scheduled for low-to-moderate-risk elective surgeries under general anaesthesia. Our exclusion criteria were (i) patients with cognitive impairment, (ii) patients with severe visual impairment (registered blind), (iii) patients with hearing impairment, (iv) patients with incomplete clinical data, and (v) patients with morbid obesity (body mass index, BMI > 35 kg/m^2^). As there was limited evidence on virtual airway and anaesthetic assessment at the time of this study in the cohort with morbid obesity, such patients were excluded because of a theoretically increased risk of difficult ventilation and/or intubation.

### 2.3. Study Design and Conduct

We conducted an observational longitudinal pilot study at a tertiary hospital in Singapore (Sengkang General Hospital, SKH) between July 2021 and April 2022. In total, 116 patients were recruited into the study, and video consultations were conducted within four weeks of the scheduled surgeries. Patients received appointment details through text messages and received a phone call reminder one day before their scheduled appointment. A select group of anaesthesiology trainees in the SKH anaesthesiology department with at least two years of anaesthesiology experience conducted the virtual consultations using the Zoom video conferencing platform (Zoom Video Communications Inc., San Jose, CA, USA). All hospital computers used for video consultations were equipped with the Zoom video conferencing software (version 5.2.0, 2020). End-to-end encryption was implemented on password-protected computers connected to the hospital’s secured networks to ensure data security. Virtual airway evaluation was performed via camera-enabled devices (smartphones or computers). Video recording functions were disabled to protect patients’ privacy and details of the video consultations were documented in the electronic medical records by the attending doctor. The same anaesthesiology trainees performed a face-to-face airway assessment on these patients on the day of the surgery as part of the pre-surgical anaesthetic evaluation in routine care. A standard anaesthesia induction technique was adopted, including administration of muscle relaxants (either rocuronium or atracurium) if intubation was required. Drug dosages were calculated based on the patient’s body weight. All intra-operative findings were recorded as mentioned above.

We evaluated five airway screening tests for difficult airways: MS, ULBT, TMD, MOT, and neck mobility. A potentially difficult airway was defined as having one or more of the following present: ULBT class III, MS 3 or 4, TMD less than five finger breadths, mouth opening less than three finger breadths, or limited neck mobility.

### 2.4. Data Collection

Patient demographics, including age, gender, ASA status, BMI, and airway assessment findings, were collected. Age, height, and weight were recorded as continuous variables. Gender, ASA status, MS, ULBT, mouth opening ≤ 3 finger breadths, TMD ≤ 5 finger breadths, and poor neck mobility (yes/no) were recorded as categorical variables. Where finger breadth assessments were made, the patients’ fingers were used during the virtual consultation. Intra-operative airway management information was also recorded, including details of airway equipment (e.g., direct or video laryngoscope), number of attempts required for successful device placement (e.g., supraglottic airway device or tracheal tube), and any difficulty encountered. Adverse events such as airway trauma (e.g., bleeding or oedema), desaturation, and dental injuries were monitored, but none were reported.

### 2.5. Data Analyses

MedCalc V.19.1.5 was used for statistical analyses. Variables were tested for normalcy using the Kolmogorov–Smirnov method [13]. Parametric data were reported as mean and standard deviation. Non-parametric data were reported as median with interquartile ranges. Bivariate correlation between virtual and face-to-face assessments was tested using the phi coefficient (Φ). To test for multivariate correlations between virtual and face-to-face assessments, the Goodman and Kruskal’s gamma (γ) coefficient was used [14]. The γ coefficient measures the association between two categories of variables with multiple ordinal data (ranks), e.g., Mallampati grades between virtual and face-to-face assessment. The phi coefficient (φ) was used to measure associations between two categories of variables with bivariate data, e.g., normal neck range of movement in virtual and face-to-face assessments: Yes/No [15]. 

Logistic regression analysis was performed to identify critical parameters in airway assessment tests that predict a PDA. An abnormal airway detected at the face-to-face assessment was used as the dependent variable (Yes = 1). For independent variables, age, weight, height, oxygen saturation, systolic blood pressure, and diastolic blood pressure were coded as non-categorical variables. The ASA grade (1,2,3,4,5,6), virtual MS (1,2,3,4), ULBT (1,2,3), mouth opening > 2 fingers (Yes = 1), BMI class (<25 = 0, 25–30 = 1, 30–35 = 2), and neck range of movement (abnormal = 1) were coded as categorical variables. Backward selection was used [16]; variables with *p* < 0.05 were included, and variables with *p* > 0.1 were removed from the model. 

Receiver operating characteristic (ROC) plots were generated to assess each tool’s overall performance, and the AUROC scores (C-statistic) were reported [17]. Two-sided tests were used, and the alpha was set at 0.05. 

## 3. Results

### 3.1. Demographics of Patients

Of the 116 patients recruited during the 10-month study period, 94 (81.0%) completed the study. Twenty-two patients dropped out due to surgery cancellations or rescheduling, including two patients who underwent conversion to physical consultation due to technical difficulties with the videoconferencing platform. In addition, 56% of patients were classified as ASA 1, and 12% had a BMI greater than 30 kg/m^2^ (obese). Although virtual and physical assessments identified difficult airways in 22.3% and 24.5% of patients, respectively, there were no actual cases of a “cannot intubate, cannot ventilate” scenario on the day of surgery (this may have been due to contingency planning). The patient demographics are summarised in Table 1.

### 3.2. Evaluating the Concordance of Virtual Versus Face-to-Face Airway Assessment

During the virtual consultation, the ULBT was successfully performed in 93 (98.9%) patients, of which 90 (96.7%) had upper lip bite class I and II. One participant could not perform the ULBT despite repeated instructions and demonstrations. During the physical assessment, this participant had class 3 ULBT. As part of the airway assessment, the presence of loose dentition was explicitly asked about during the virtual consultation, and a dental referral was initiated if present. Results of the airway screening tests during the virtual assessment and, subsequently, the preoperative in-person assessment are presented in Table 2. Specifically, there was strong concordance between virtual and face-to-face assessment results for the ULBT, MOT, and neck mobility (i.e., the same anaesthesiology trainees reported identical results in virtual and face-to-face assessments most of the time). The correlation of Mallampati scores was only moderately strong between virtual and face-to-face assessments, i.e., some MSs were different between virtual and face-to-face assessments (γ = 0.46). Thyromental distance and loose dentition assessments showed no relationship between virtual and face-to-face assessments (i.e., most of these results were dissimilar).

In patients with a BMI of 30 to 35 kg/m^2^, obesity alone was not significantly associated with PDAs. However, logistic regression showed that being overweight (BMI > 25 kg/m^2^) may be a predictor (see below, *p* = 0.077, but this may fall if sample size increases).

### 3.3. Performance of Virtual Airway Assessment Tools

Multiple logistic regression analysis demonstrated that the virtual MS (vMS), virtual upper lip bite test (vULBT), and elevated BMI (>25 kg/m^2^) are potential predictors of difficult airways (see Table 3).

**Figure 1:**
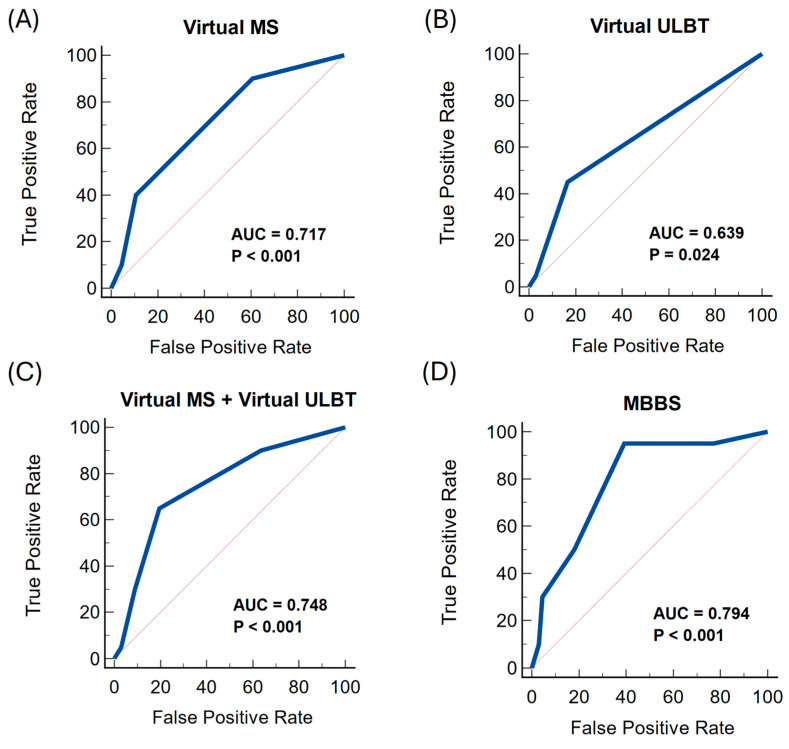
ROC curves of various virtual airway assessment methods compared against the gold standard (in-person assessment findings).

Notably, as individual tests to predict difficult airways, the vMS in Figure 1A and the vULBT in Figure 1B showed suboptimal performance only: AUC 0.72 and AUC 0.64, respectively. When used together, vMS and vULBT performed significantly better; AUC 0.75 (Figure 1C). A summation of the v**M**S (1/2/3/4), vUL**B**T class (1/2/3), and patient **B**MI category (0/1/2) into a single **S**core, termed the “MBBS”, showed the best performance: AUC 0.79 (Figure 1D). Table 4 illustrates the other performance statistics of the various virtual airway assessment tests.

## 4. Discussion

Our study found that virtual airway assessment using standard airway assessment tools detects PDAs poorly; the sensitivity of vMS and vULBT was only 40% and 33.3%, respectively. Coupled with their low AUC scores (reported above), our data suggest that these tests are unsuitable when used virtually. As previously highlighted by a panel of Cochrane reviewers [3], high sensitivity is crucial for identifying a PDA preoperatively, allowing for appropriate preparation and management.

Multiple studies have identified obesity as an independent risk factor for difficult airway management, in line with the NAP 4 report [1,2,18,19]. When we combined obesity (i.e., elevated BMI) with several tests, the resultant MBBS score exhibited a sensitivity of 95.0% and an AUC of 0.79 in a conservatively sized pilot study [20]. These promising results suggest that the MBBS score may be used virtually in clinical practice, and further studies in a larger cohort of patients, recruited in multiple centres, are justified. This will be the direction of our future work.

The MS is a commonly used airway assessment tool for evaluating the tongue and pharyngeal size and their relationship. Class 3 or 4 is associated with difficult intubation [21]. However, in two meta-analyses, the MS had only a moderate ability to predict difficult airways, with a reported sensitivity of 51% [22,23]. In our study, low levels of lighting and/or low camera resolution on the patient’s end of the Zoom platform may have limited the visualisation of the oropharyngeal airway structures, leading to discrepancies between virtual and face-to-face MS. Inter-rater variability was reduced because the same group of anaesthesiology trainees performed the virtual and physical airway assessment on the same group of patients. Nonetheless, the sensitivity of the vMS in predicting abnormal airways was low (40.0%) but similar to that in the existing literature.

The ULBT evaluates mandibular movement by asking the patient to bite the upper lip as far as possible with the lower incisors. Class III is an indicator of a difficult airway. In a recent systematic review of the bedside UBLT in predicting difficult airways, its sensitivity was more than 70% in 11 out of 27 studies [24]. A recent virtual airway assessment study by Zhao et al. reported fair and good inter-rated agreement between anesthesiologists in person airway evaluations compared with medical students and anesthesiologists’ virtual airway assessments respectively [25]. Airway assessment tests used were similar to in our study (MS, thyromental distance, and mouth opening) but the ULBT was excluded. In addition, test performances were also not reported. There is a paucity of evidence of ULBT utilisation in the virtual setting, and in our study, the sensitivity of the virtual ULBT was only 33.3%. Notably, there is considerable heterogeneity in the sensitivity and specificity of bedside airway screening tests studied to date [26]. 

Technological equipment and familiarity with videoconferencing platforms may significantly influence the accuracy of virtual consultations. In our study, there were no restrictions on minimal technological proficiency or device specifications for patients. Patients used their own available devices, including mobile phones, tablets, and desktop computers. Some challenges encountered during our study included poor internet connectivity, low lighting conditions, and suboptimal video or image quality, thereby reducing the accuracy of airway assessment scoring. These observations are consistent with previous publications [7,27,28]. To circumvent device variation and performance for future studies, a possibility would be to standardise equipment, e.g., the use of hospital smartphones loaded with a pre-installed app that links the smartphone’s camera and its light source (flash bulb) directly to Zoom or another web conferencing platform. This may be loaned to patients and returned on the day of surgery.

This study had several limitations. Firstly, as this was a pilot study, the sample size was limited to 94 patients who were considered relatively low risk for the study. Male gender and obesity are known risk factors for failed or difficult intubation [29]. The predominantly male population (77.7%) and exclusion of patients with morbid obesity (BMI >35 kg/m^2^) in our study may affect generalizability, and further validation of the MBBS score in virtual assessment is required in larger and broader populations. Secondly, although PDAs were identified, there were no instances of difficult airway management on the day of surgery. This may also have been due to contingency planning, e.g., using video laryngoscopes (McGrathTM is the default option in our institution) and supraglottic airway devices early if any difficulty was encountered. This study deliberately excluded patients with class 2 and class 3 obesity. This was because, as a pilot study, we did not want to expose high-risk patients to even more risk. We will conduct further studies on high-risk patient groups (e.g., patients with known difficult airways, obstetric populations, and populations with obesity) through larger trials to help define the predictive value of the MBBS in difficult intubation. In patients at risk of PDAs (e.g., head and neck pathology), consideration of virtual endoscopy, coupled with clinical history and computerised tomography imaging, may enhance airway assessment [30].

## 5. Conclusions

Existing bedside airway assessment tests perform poorly when used to screen for PDAs virtually and when used in isolation. Combining patient risk factors and multiple tests in virtual assessments can increase the detection and diagnostic accuracy of PDAs, allowing for the safe incorporation of virtual screening into clinical practice. Further large-scale studies to validate the MBBS for clinical use are required and justified.

## Figures and Tables

**Table 1:** Demographics of patients who had virtual and face-to-face airway assessments.

Variable	Results
Age (years)	Median = 37, IQR 30–46
Gender	Male 77.7% (73/94)
	Female 22.3% (21/94)
BMI (kg/m^2^)	24.8 ± 3.9
Normal airway at virtual assessment	Yes = 77.7% (73/94)
(“No” = potentially difficult airways)	No = 22.3% (21/94)
Normal airway at pre-op assessment	Yes = 75.5% (85/94)
(“No” = potentially difficult airways)	No = 24.5% (23/94)

IQR = interquartile range; BMI = body mass index. Median and IQR were reported when data did not follow a normal distribution.

**Table 2:** Virtual assessment versus face-to-face assessment results. γ = Goodman and Kruskal’s gamma coefficient; Φ = phi coefficient.

Parameters Assessed	Virtual Assessment	Face-to-Face Assessment	Coefficient	*p* Value
Upper lip bite test				
Class 1	75.5% (71/94)	83.0% (78/94)	0.75	0.001
Class 2	21.3% (20/94)	16.0% (15/94)	(γ)	
Class 3	3.2% (3/94)	1.0% (1/94)		
Mouth opening > 2 fingers				
Yes	96.8% (91/94)	100% (94/94)	0.94	<0.001
No	3.2% (3/94)	0	(φ)	
Thyromental distance > 3 fingers				
Yes	92.6% (87/94)	90.4% (85/94)	0.05	0.660
No	7.4% (7/94)	9.6% (9/94)	(Φ)	
Full range of neck movement				
Yes	100% (94/94)	100% (94/94)	1.0	<0.0001
No	0	0	(Φ)	
Mallampati score				
Class 1	32.6% (28/86)	42.6% (40/94)	0.46	0.0001
Class 2	50.0% (43/86)	34.0% (32/94)	(γ)	
Class 3	11.6% (10/86)	19.2% (18/94)		
Class 4	5.8% (5/86)	4.2% (4/94)		
Loose dentition				
Yes	1.1% (1/94)	1.1% (1/94)	−0.01	0.830
No	98.9% (93/94)	98.9% (93/94)	(Φ)	

**Table 3:** Results of multiple logistic regression. AUROC of model = 0.77 (95% CI: 0.66 to 0.0.85).

Variables	B	SE	Wald	*p*-Value	OR	95% CI (Lower)	95% CI (Upper)
vMS	2.017	0.780	6.684	0.010	7.514	1.629	34.667
vULBT	1.383	0.634	4.768	0.029	3.989	1.152	13.809
BMI > 25	1.079	0.610	3.125	0.077	2.940	0.889	9.721

**Table 4:** Performance statistics of standard airway assessment tests when used virtually. The MBBS had the highest sensitivity, AUROC, and negative predictive value.

Tests and Performance Statistics	Virtual MS (Class ≥ 3)	Virtual ULBT (Class 3)	Virtual MS + ULBT (Score ≥ 4)	Virtual MS + ULBT + BMI (MBBS) (Score ≥ 4)
Sensitivity	40.0%	33.3%	85.0%	95.0%
(95% CI)	(19.1% to 64.0%)	(0.8% to 90.6%)	(62.1% to 96.8%)	(75.1% to 99.9%)
Specificity	89.4%	75.8%	66.7%	60.6%
(95% CI)	(79.4% to 95.6%)	(65.7% to 84.2%)	(54.0% to 77.8%)	(47.8% to 72.4%)
AUROC	0.65	0.55	0.75	0.78
(95% CI)	(0.54 to 0.75)	(0.44 to 0.65)	(0.65 to 0.84)	(0.68 to 0.86)
Positive Likelihood Ratio	3.8	1.4	2.6	2.4
(95% CI)	(1.6 to 9.1)	(0.3 to 7.1)	(1.7 to 3.8)	(1.8 to 3.3)
Negative Likelihood Ratio	0.7	0.9	0.2	0.1
(95% CI)	(0.5 to 1.0)	(0.4 to 2.0)	(0.1 to 0.6)	(0.0 to 0.6)
Positive Predictive Value	53.3%	4.3%	43.6%	42.2%
(95% CI)	(32.1% to 73.4%)	(0.9% to 19.0%)	(34.4% to 53.2%)	(34.8% to 50.1%)
Negative Predictive Value	83.1%	97.2%	93.6%	97.6%
(95% CI)	(77.3% to 87.7%)	(93.9% to 98.7%)	(83.6% to 97.7%)	(85.4% to 99.6%)
Accuracy	77.9%	74.5%	70.9%	68.6%
(95% CI)	(67.7% to 86.1%)	(64.4% to 82.9%)	(60.1% to 80.2%)	(57.7% to 77.2%)

## Data Availability

The original contributions presented in this study are included in the article. Further inquiries can be directed to the corresponding author.
